# Adsorption of tetracycline on polyvinyl chloride microplastics in aqueous environments

**DOI:** 10.1038/s41598-023-44288-z

**Published:** 2023-10-20

**Authors:** Mohaddeseh Zahmatkesh Anbarani, Aliasghar Najafpoor, Behnam Barikbin, Ziaeddin Bonyadi

**Affiliations:** 1 Student Research Committee, Mashhad University of Medical Sciences, Mashhad, Iran; 2 Department of Environmental Health Engineering, School of Health, Mashhad University of Medical Sciences, Mashhad, Iran

**Keywords:** Environmental sciences, Chemistry, Engineering

## Abstract

Microplastics (MPs), as carriers of organic pollutants in the environment, have become a growing public concern in recent years. Tetracycline (TTC) is an antibiotic that can be absorbed by MPs and have a harmful effect on human health. Therefore, this study was conducted with the aim of investigating the adsorption rate of TTC onto polyvinyl chloride (PVC) MPs. In addition, the adsorption mechanism of this process was studied using isothermal, kinetic, and thermodynamic models. For this purpose, experimental runs using the Box-Behnken model were designed to investigate the main research parameters, including PVC dose (0.5–2 g/L), reaction time (5–55 min), initial antibiotic concentration (5–15 mg/L), and pH (4–10). Based on the research findings, the highest TTC adsorption rate (93.23%) was obtained at a pH of 10, a contact time of 55 min, an adsorbent dose of 1.25 g/L, and an antibiotic concentration of 10 mg/L. The study found that the adsorption rate of TTC followed the pseudo-second-order and Langmuir models. Thermodynamic data indicated that the process was spontaneous, exothermic, and physical. Increasing ion concentration decreased TTC adsorption, and distilled water had the highest adsorption, while municipal wastewater had the lowest adsorption. These findings provide valuable insights into the behavior of MPs and organic pollutants, underscoring the importance of conducting additional research and implementing measures to mitigate their detrimental effects on human health and the environment.

## Introduction

Plastics are used in everyday life due to their advantages, such as high durability, flexibility, low cost, and resistance to oxidation. This has led to a significant increase in the production of this product worldwide. So far, the amount of plastic has reached approximately 1.5 million tons since 1950. It is projected that this figure will increase to 445.25 million tons by 2025^1^. Recently, microplastics (MPs) have been investigated as potential byproducts of plastic degradation, which is significant due to their detrimental effects on the environment and living organisms^2^. The annual release of approximately 8 million tons of plastic into marine and freshwater environments has resulted in the presence of MPs in these waters, posing a threat to the lives of aquatic organisms^3^. These particles, which are known to be smaller than 5 mm in size, enter the environment in two primary and secondary forms. They are mainly created from sources such as cosmetics, machine clothes containing fibers, cleaning products used in scrubbers, textiles, personal care products, and the degradation of large plastics in the environment^4^.

MP particles can prevent photosynthesis and growth by penetrating algal cells. By creating a false sense of satiety, MPs disturb the nutrition of algae^5^. Also, these pollutants enter the bodies of crabs. Due to the difficulty of removing MPs from their bodies, the MPs remain in their intestines for approximately 14 days^6^. In addition, MPs can absorb various pollutants such as antibiotics, heavy metals, drugs, and pesticides due to their polarity, crystallinity, and wide pores. The adsorption of organic pollutants by MPs increases their toxicity and their tendency to bioaccumulate in the environment. Smaller MPs have a higher adsorption capacity due to their larger surface area^7,8^. Researchers recently found that polypropylene (PP), polyvinyl chloride (PVC), polystyrene (PS), polyamide (PA), and polyethylene (PE) are common types of MPs in aquatic environments^9^. PVC is used in cable insulation, window frames, and pipes due to its advantages, such as low cost, durability, and favorable physical and chemical properties. Products made with PVC have a longer lifespan compared to other plastics. However, under the influence of environmental factors, PVC may turn into small particles of MPs. PVC MPs pose significant risks to human health, particularly in terms of mutagenesis and carcinogenesis. The small size and chemical composition of these MPs enable them to penetrate tissues and cells, interfering with cellular mechanisms and the growth of cancerous tumors. Accumulation of PVC MPs in various organs and tissues can contribute to chronic inflammation, cellular damage, and an increased risk of cancer^10^.

Antibiotics are a class of medicinal compounds that are widely used in the fields of veterinary medicine, agriculture, and human medicine to treat diseases^11^. Contamination of aquatic environments with antibiotics is considered a serious problem. According to the report of the World Health Organization, these pharmaceutical compounds are responsible for 25% of water pollution worldwide^12^. Tetracycline (TTC) was considered one of the antibiotics used in 1948 to treat various microbial infections, including chlamydia, anthrax, syphilis, cholera, Lyme disease, and pneumonia^13^. This antibiotic is the second most commonly used antibiotic in the world. TTC is a polar and ionizable antibiotic that contains several functional groups, including amino, carbonyl, and hydroxy groups^14^. The entrance of TTC into aquatic ecosystems has raised concerns about its detrimental effects on the environment and human health^15^. Antibiotics often contaminate various water environments, including underground water, surface water, soil, and even drinking water, due to urban sewage treatment plants and agricultural runoff^16^. Although only a small portion of TTC is absorbed by the human body, the majority is excreted. Consumption of drinking water contaminated with TTC can lead to gastrointestinal irritation, diarrhea, and vomiting. In addition, TTC can be absorbed by bones and inhibit bone growth^17^. Yu et al.^18^ reported that the adsorption of TTC by MPs is influenced by the surface properties of MPs and the chemical properties of the aqueous solution play an important role in the adsorption of TTC. In a study, it has been proven that bisphenol analogues are adsorbed onto PVC through mechanisms such as non-covalent bonds, electrostatic forces, hydrogen bonding, and hydrophobic interactions^19^. In another study, it was discovered that norfloxacin adsorbed onto PP, PS, PVC, and PE MPs through π–π bonds, H-bonds, van der Waals interactions, and polar-polar interactions^7^. Wang et al.^20^ investigated the effect of polyethylene on pesticide residues in aqueous environment.

MPs pose health and environmental risks due to their potential toxicity to the environment, inability to biodegrade, and capacity to adsorb chemical pollutants. The coexistence of MPs and organic pollutants, such as antibiotics, in the aquatic environment exacerbates toxicity and stress on the natural aquatic ecosystem^21^. Therefore, this study was conducted with the aim of investigating the factors that affect the adsorption capacity of TTC on PVC from an aqueous medium. Furthermore, the characteristics of TTC and PVC were analyzed using EDX, FTIR, and FESEM.

## Materials and methods

### Chemicals

PVC with a grade of 57, dimensions of ≤ 85 µM and a pore volume of 0.05–0.2 mL/g was supplied by Abadan Petrochemical Company, Iran. TTC with a purity of 99% was provided by Sinadaro Company, Iran. Other chemicals such as CaCO_3_, MgSO_4_, NaHCO_3_, Na_2_SO_4_, C_8_H_5_KO_4_, NaCl, HCl, and NaOH were purchased from Merck Company, Germany. Distilled water was used in all stages of this experiment.

### Characterization techniques

Field emission scanning electron microscopy (FESEM) imaging was used to observe the changes in surface morphology of particles. The FESEM analysis was carried out using a Supra 55 electron microscope manufactured by Carl Zeiss in Germany. To determine the chemical composition, bonds, and functional groups of the PVC before and after adsorption, Fourier transform infrared spectrometer (FTIR) analysis was performed using a PerkinElmer spectrometer, specifically the FT-IR/NIR FRONTIER model. Furthermore, energy-dispersive X-ray (EDX) analysis was employed to determine the elemental composition of the samples. This analysis was conducted using an Oxford device connected to a JEOL-JSM-5600 SEM.

### Batch adsorption experiment

**Figure 1:**
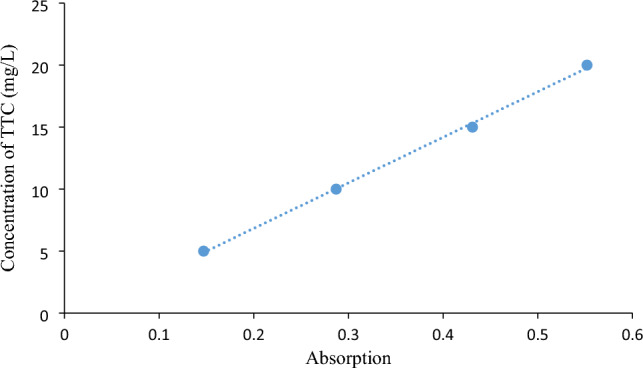
TTC calibration curve at concentrations ranging from 5 to 20 mg/L.

At the beginning of the experiment, a TTC stock solution was prepared with a concentration of 100 mg/L. The purification of TTC was conducted using PVC in a glass reactor. The reaction mixture, which consisted of 50 cc, was subjected to various variables, including the initial antibiotic concentration (5–15 mg/L), solution pH (4–10), adsorbent dose (0.5–2 g/L), and reaction time (5–55 min). The purification process was performed on a magnetic shaker at a speed of 300 rpm. After completing the experiment, 10 mL of the reaction mixture were taken and centrifuged at 3000 rpm for 10 min. The suspension was filtered using a 0.22 μm filter. To measure TTC, first, concentrations of 5–20 mg/L TTC were prepared, and a standard curve was plotted. Then, the concentration of TTC in the sample was determined using a UV–Vis spectrophotometer at a wavelength of 356 nm. The final adsorption rate of TTC in the samples was calculated using the following formula:

1 $${\text{TTC}}\;{\text{adsorption\% }} = \frac{{\left( {{\text{C}}_{0} - {\text{Ce}}} \right) \times 100}}{{{\text{C}}_{0} }}$$

where “C_0_” is the initial concentration of TTC (mg/L) and “C_e_” is the equilibrium concentration of TTC (mg/L).

2 $${\mathrm{q}}_{\mathrm{e}}=\frac{{(\mathrm{C}}_{0}-{\mathrm{C}}_{\mathrm{e}})}{\mathrm{m}}\times \mathrm{V}$$

where “m” represents the mass of PVC (g), and “V” represents the volume of the reaction mixture (L). Figure 1 shows the TTC calibration curve at concentrations ranging from 5 to 20 mg/L.

### Study matrix

As previously mentioned, a statistical technique was utilized to encompass the entire process of designing, modeling, and optimizing the TTC adsorption study. The adoption of the Box–Behnken design (BBD) allowed for the establishment of a model that aimed to maximize the adsorption of TTC onto PVC. The BBD methodology is a more advanced alternative to the traditional “one factor at a time” approach because it enables the determination of optimal conditions and the interaction effects among variables. BBD employs a three-level rotatable or nearly rotatable quadratic design, where variables are studied at their midpoints (± 1) and the center point (0)^22^. Table 1 shows the ranges and values of studied factors. In this work, 29 experimental runs, based on the BBD design, were conducted to study the four process factors. The data obtained in the experiments were analyzed using a second-order polynomial model to find the linear and quadratic effects of factors on TTC adsorption. The ANOVA analysis uses the following equation (Eq. 3) to describe the process

**Table 1:** Range and levels of the main factors used for TTC adsorption.

Factor	Code	Variable level		
		− 1	0	+ 1
TTC conc. (mg/L)	A	5	10	15
Time (min)	B	5	30	55
pH	C	4	7	10
Dose of PVC (g/L)	D	0.5	1.25	2

3 $$\mathrm{Y}={\upbeta }_{0}+\sum_{\mathrm{i}=1}^{\mathrm{k}}{\upbeta }_{i}{\mathrm{X}}_{\mathrm{i}}+\sum_{\mathrm{i}=1}^{\mathrm{k}}{\upbeta }_{ii}{\mathrm{X}}_{\mathrm{i}}^{2}+\sum_{\mathrm{i}=1}^{\mathrm{k}-1}1\sum_{j-1}^{\mathrm{k}}{\upbeta }_{ij}{\mathrm{X}}_{\mathrm{i}}{\mathrm{X}}_{\mathrm{j}}+\upvarepsilon$$

In the Eq. (3), Y is the response (MG removal), X_i_ and X_j_ are coded factors, β_0_ is a constant level, β_i_, β_ii_, and β_ij_ are the first-order effects, second-order effects, and interaction impacts regression coefficients and $\upvarepsilon$ is the random error^23^.

### Adsorption isotherm and kinetic studies

The investigation of factors affecting the adsorption rate necessitates the examination of kinetics. The adsorption isotherm and kinetics were analyzed in the presence of various factors, including TTC concentration ranging from 2 to 16 mg/L, contact time ranging from 10 to 90 min, a fixed pH of 7, and a prepared PVC concentration of 1 g/L. To predict the isotherm mechanism, the Langmuir, Freundlich, and Temkin models were used. Additionally, the study involved the application of kinetic models, including the pseudo-first-order kinetic model, pseudo-second-order kinetic model, and intra-particle diffusion model^24^.

### Langmuir isotherm

E Langmuir model proposes that adsorption occurs on a surface that is homogeneous, uniform, and has uniform energy. The Langmuir isotherm is presented as follows^25^:

4 $$\frac{Ce}{{qe}} = \frac{1}{{q_{m} \times K_{L} }} + \frac{Ce}{{q_{m} }}$$

In this formula, q_max_ represents the maximum sorption capacity (mg/g), q_e_ represents the equilibrium sorption capacity, and K_L_ represents the Langmuir constant (L/mg).

### Freundlich isotherm

The Freundlich model has a contrasting nature compared to the Lang model. In this model, the adsorption process occurs on a surface with distinct characteristics, including variations in energy distribution, heterogeneity, and non-uniformity. The Freundlich isotherm is shown as Eq. (5)^26^:

5 $$lnq_{e} = lnk_{F} + \frac{1}{n}lnCe$$

where qe is the equilibrium sorption capacity, and n and KF (l/g) are the constants of the Freundlich model.

### Temkin isotherm

Temkin's model focuses on the interaction occurring on the suace ansuggests that the connection between adsorbed molecules and their interactions diminishes linearly. The equation for Temkin's isotherm, as stated in reference^27^, can be expressed as follows:

6 $$q_{e} = B_{1} ln_{.} K_{t} + B_{1} ln_{.} C_{e}$$

where B_1_ is the activity coefficient (J/mol) and K_t_ is the constant of Tamkin model.

### Kinethics models

#### Pseudo-first-order

Cording to this model, the amount of adsorbent directly affects the rate of adsorption at the interface between solid and fluid surfaces. The pseudo-first-order kinetics can be represented as follows^28^:

7 $$\log \left( {q_{q} - q_{t} } \right) = {\text{log}}\, q_{e } - \frac{{K_{1} }}{2.303} \cdot t$$

where q_t_ and q_e_ (mg/g) represent the adsorption capacity of TTC at the desired time and under equilibrium conditions, respectively. The constant K_1_ (min^−1^) represents the rate constant for the adsorption rate in pseudo-first-order kinetics.

#### Pseudo-second-order

This model proposes that the process is governed by chemisorption, which occurs when electrons are shared or covalent forces are present between the adsorbent and adsorbed molecules. Pseudo-second-order kinetics can be represented as^29^:

8 $$\frac{t}{{q_{t} }} = \frac{1}{{k_{2} q_{e}^{2} }} + \frac{1}{{q_{e} }} \cdot t$$

where k_2_ is the rate constant for pseudo-second-order kinetics (g/mg min)

### Intraparticle diffusion

The intraparticle diffusion model determines which factors may be limiting the reaction rate and identifies the diffusion mechanism. Intra-particle diffusion kinetics is expressed as^30^:

9 $$q_{t} = k_{p} \cdot t^{0.5} + C$$

where k_p_ is the constant of intraparticle diffusion rate (mg/g min^1/2^), and C is a constant of the intraparticle diffusion model that is associated with the thickness of the boundary layer. The intraparticle diffusion model investigates whether the adsorption process is controlled by intraparticle diffusion or boundary layer diffusion.

### Thermodynamic

To enhance our understanding of the adsorption mechanism of TTC on PVC, we conducted a thermodynamic analysis.

The Van’t Hoff equation (Eq. 10) was utilized to express the thermodynamic calculation^31^:

10 $$\Delta {\text{G}}^\circ = - {\text{RTlnK}}$$

In this equation, ΔG represents the Gibbs free energy change in kJ/mol, K denotes the equilibrium constant, R is the gas constant (8.314 J/mol K), and T represents the temperature in Kelvin (K).

To calculate the spontaneous rate of a process, the formula used is the change in Gibbs free energy (ΔG°), which can be determined by the following equation^32,33^:

11 $${\Delta {\text G}}{^\circ } = {\Delta {\text H}}{^\circ } - {{{\text{T}}\Delta {\text S}}}{^\circ }$$

ΔH represents the enthalpy change (kJ/mol), and ΔS° represents the entropy change (kJ/mol^−1^). This relationship is expressed by the following formula^34^:

12 $$\ln {\text{K}} = - \frac{{{\Delta {\text H}}{^\circ }}}{{{\text{RT}}}} + \frac{{{\Delta {\text S}}{^\circ }}}{{\text{R}}}$$

## Results and discussion

### Characterization

**Figure 2:**
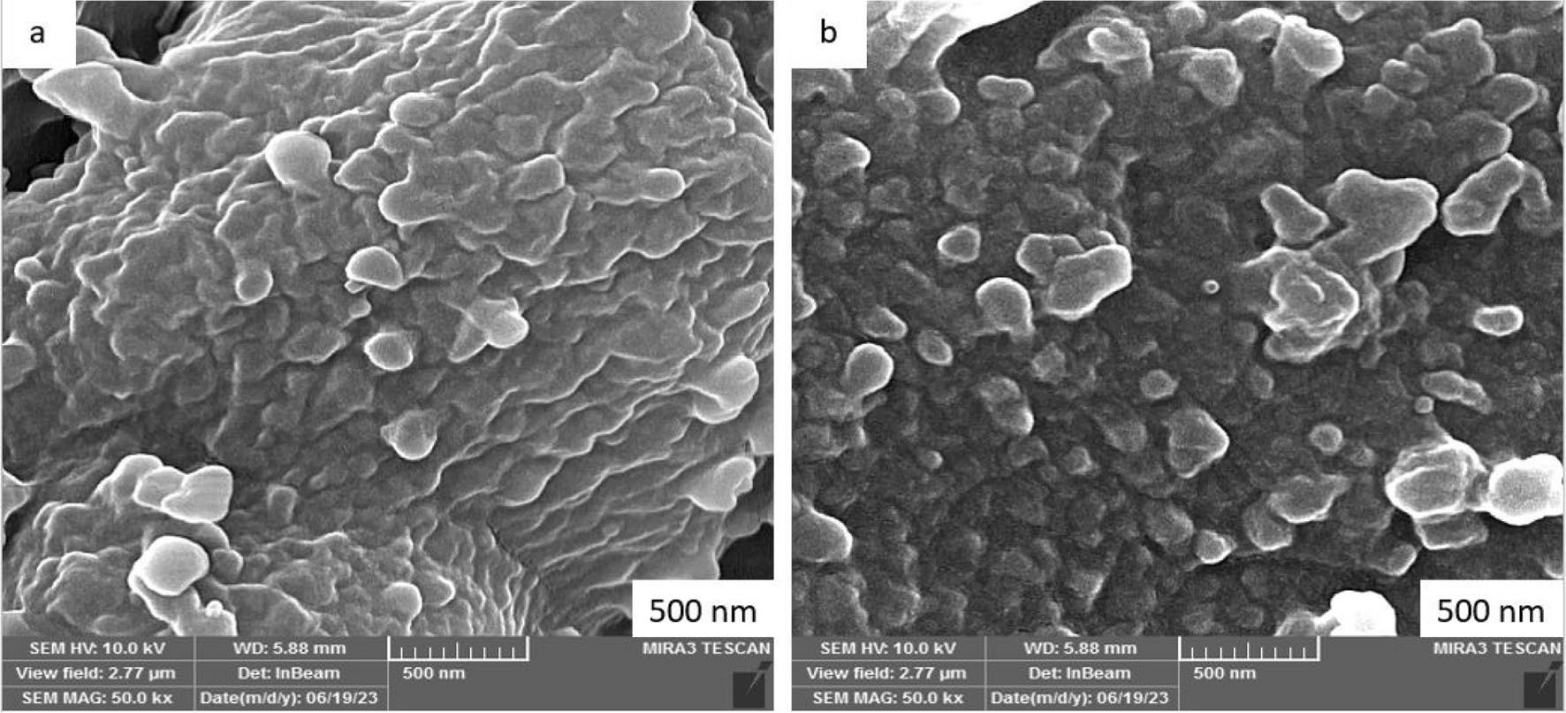
FESEM images of PVC before and after TTC adsorption.

FESEM is the best method for magnifying and examining the surface structure, as well as observing features such as roughness, porosity, and surface cracks. This information is important for understanding how the structure of the adsorbent surface affects the adsorption process^35^. Figure 2a shows the images of PVC before and after the adsorption process. According to Fig. 2a, PVC has a non-uniform surface and consists of spherical chains. In addition, it has large valleys and grooves on its surface. In fact, these characteristics of the PVC surface represent a positive aspect, as they provide a large surface area and active sites for adsorption. PVC can adsorb more pollutants than other MPs for several reasons. First, PVC has a larger surface area compared to other MPs. As a result, there are more adsorption sites on its surface^7^. Second, MPs can form polar bonds with polar pollutants. Additionally, PVC has a high degree of crystallinity, which allows MPs to exist in a glass state. This glass state makes it easier for MPs to encapsulate pollutants^35^. Yu et al.^4^ inferred that PVC may have more adsorption sites than other MPs due to its uneven surface and internal wrinkles. As can be seen from Fig. 2b, the seams and cracks on the PVC surface are slightly reduced after TTC adsorption. This confirms that the adsorption of TTC towards MPs has been successful. Hu et al.^36^ confirmed that PVC has a strong polarity and can adsorb TTC through polar interactions. In another study, it was found that polar MPs have a better ability to absorb organic pollutants compared to non-polar MPs^5^.

**Figure 3:**
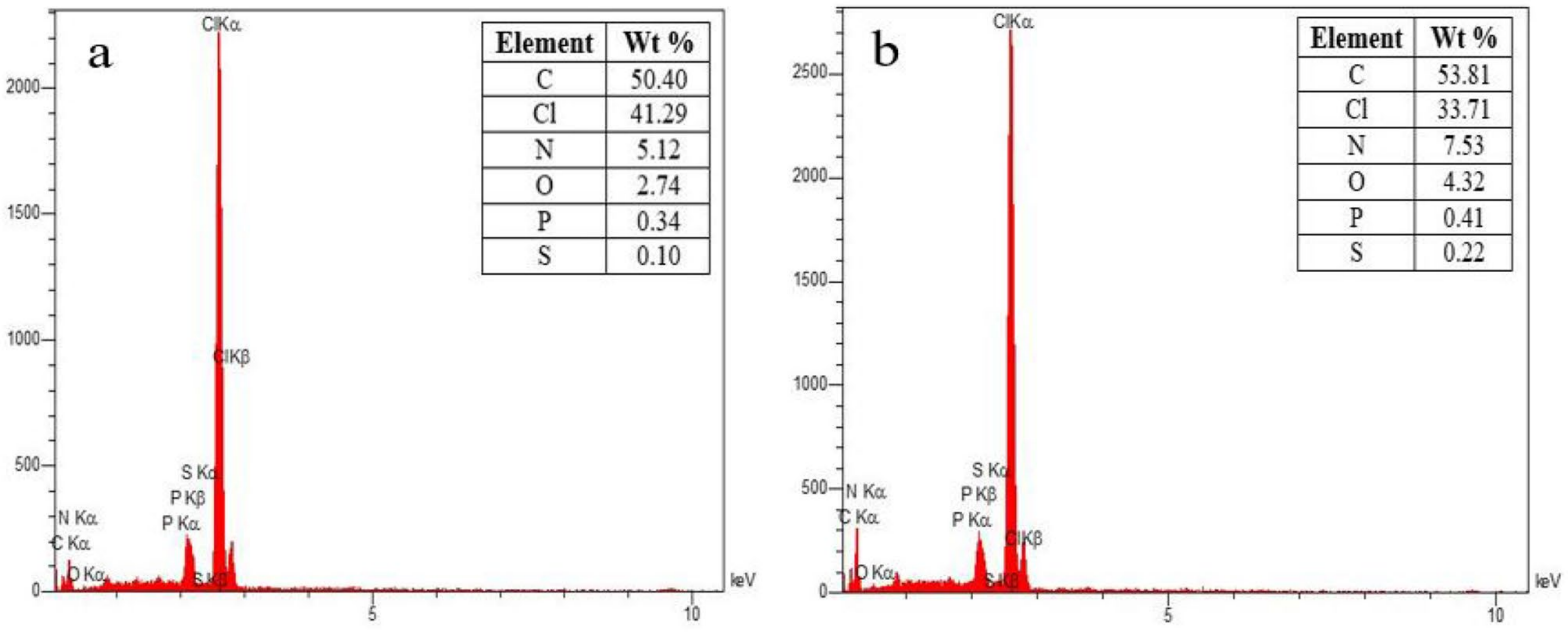
EDX spectrum of PVC (**a**) before and (**b**) after TTC adsorption.

EDX analysis determined the amounts of elements in PVC before and after TTC adsorption. Figure 3a,b shows the elements in PVC before and after the adsorption process. According to Fig. 3a, the concentrations of C, Cl, N, O, P, and S before the adsorption process were 50.40%, 41.29%, 5.12%, 2.74%, 0.34%, and 0.10%, respectively.

As can be seen from Fig. 3b, the amounts of these elements changed during the adsorption process.

For example, the chlorine content decreased to 33.71%, while the amounts of other elements increased. Tetracycline is a complex molecule containing carbon (C), hydrogen (H), nitrogen (N), oxygen (O), sulfur (S), and phosphorus (P) atoms. During the absorption process, when TTC comes into contact with PVC, certain sulfur (S) and phosphorus (P) atoms from TTC molecules are transferred to the surface of PVC MPs. This leads to an increase in the concentration of S and P after the adsorption process. Also, the decrease in chlorine content (Cl) was attributed to the interaction between chlorine atoms in PVC and TTC molecules. TTC contains functional groups that can form bonds or complexes with Cl atoms. Consequently, some of the Cl content in PVC is likely to participate in these interactions, resulting in a decrease in the Cl concentration on the PVC surface.

**Figure 4:**
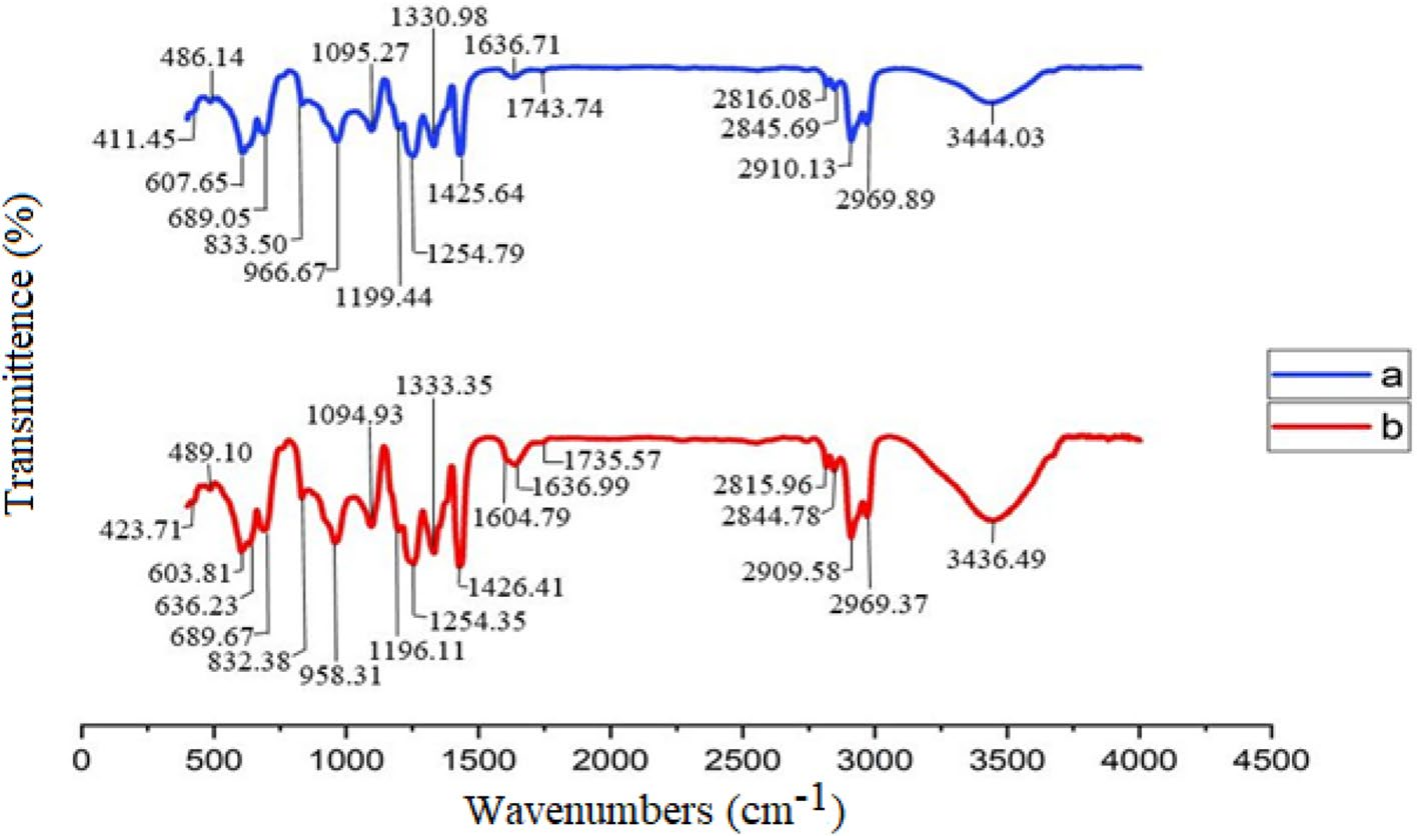
FTIR spectrum of PVC (**a**) before and (**b**) after TTC adsorption.

Figure 4a shows the FT-IR spectrum of PVC before adsorption removal. The peak at 1254.79 cm^−1^ indicates the presence of C–O stretching vibration in PVC. The peak observed at 1330.98 cm^−1^ is associated with the C–O–C bond^37^. The peak at 1425.64 cm^−1^ indicates the presence of a carbon chain in PVC. The peak at 1636.71 cm^−1^ is related to C=O bands^38^. The peak at 2845.69 cm^−1^ is attributed to the symmetric stretching of CH_2_. The –CH_2_ bonds exhibit a peak at 2910.13 cm^−1^. Asymmetrical vibrations at 2969.89 cm^−1^ are associated with C–H groups. The peak at 3444.03 cm^−1^ is related to the O–H stretching vibration^39^.

Figure 4b illustrates the FT-IR spectrum after TTC adsorption. The shift of the peak to 636.99 cm^−1^, indicating the presence of C=O groups, confirmed the strong binding between TTC molecules and PVC. The presence of chlorine in the composition of PVC can be detected by the peak at about 832 cm^−1^ in the FTIR spectrum. This peak is caused by C–Cl stretching vibration in PVC. When TTC is adsorbed on PVC, the molecules interact with the chlorine atoms in the PVC, leading to a shift of the peak from 832.5 to 832.38 cm^−1^. The C–O stretching vibration of the carboxylic acid was observed at a peak of 1254.35 cm^−1^. After TTC adsorption, the peak associated with C–O–C stretching vibration shifted to 1333.35 cm^−1^^40^. Findings indicated that after the adsorption process, the peak related to CH_2_ symmetric stretching changed to 2844.78 cm^−1^^41^. The peak associated with –CH_2_ is transferred to 2909.58 cm^−1^. The C–H bonds shifted from 2969.89 to 2969.37 cm^−1^^4^. The peak related to O–H groups was changed to 3436.49 cm^−1^. Appearing C–H and O–H groups after the adsorption process indicates that TTC molecules are connected to PVC through hydrogen bonding^36^.

### Modeling the adsorption rate of TTC

The study evaluated the impact of various intervening factors, including the PVC dose, the initial concentration of TTC, contact time, and pH, on the removal of TTC. The findings indicate the adsorption of TTC onto PVC.

The experimental findings were statistically evaluated using linear, 2FI, quadratic, and cubic models to determine the model that best represents the data. In Table 2, the adsorption rate of TTC ranged from 10.43 to 90.47%. Table 3 represents the evaluation of the statistical adequacy of the models. Based on Table 3, the quadratic model is recommended as it has the highest adjusted R^2^ value of 0.8791, indicating that it explains 87.91% of the variation in the data. Additionally, the sequential *p* value for the quadratic model is less than 0.0001, indicating that the model is statistically significant.

**Table 2:** BBD matrix for the adsorption of TTC onto PVC.

Run no	Coded variable				Removal (%)	Run no	Coded variable				Removal (%)
	A	B	C	D			A	B	C	D	
1	1	1	0	0	49.92	16	0	0	0	0	39.76
2	0	0	− 1	− 1	31.54	17	− 1	0	1	0	82.67
3	− 1	1	0	0	79.57	18	0	− 1	0	1	25.23
4	− 1	0	0	− 1	41.52	19	0	0	1	1	48.87
5	1	0	− 1	0	40.76	20	0	1	− 1	0	40.91
6	0	− 1	− 1	0	47.3	21	0	0	1	− 1	65.72
7	0	1	0	− 1	63.18	22	0	1	0	1	33.13
8	− 1	0	0	1	52.6	23	0	0	0	0	47.5
9	0	0	0	0	39.09	24	1	0	0	1	20.27
10	0	0	− 1	1	24.99	25	0	1	1	0	93.23
11	0	0	0	0	37.41	26	1	0	1	0	72.31
12	0	0	0	0	35.21	27	− 1	− 1	0	0	64.03
13	− 1	0	− 1	0	56.99	28	0	− 1	1	0	50.16
14	1	− 1	0	0	43.02	29	1	0	0	− 1	53.82
15	0	− 1	0	− 1	53.26

**Table 3:** Evaluation of statistical adequacy for models.

Source	Sequential *p* value	Lack of fit *p* value	Adjusted R^2^	Predicted R^2^
Linear	< 0.0008	0.0221	0.4542	0.2985
2FI	0.3452	0.0224	0.4817	0.0374
Quadratic	< 0.0001	0.2605	0.8791	0.6937
Cubic	0.1907	0.3958	0.9258	0.1348

Table 4 presents the coefficients for the quadratic model of TTC removal by PVC. According to Table 4, the quadratic model was fitted to the experimental data.

**Table 4:** Coefficients of estimation for the quadratic model of TTC removal by PVC.

Factor	Coefficient estimate	*df*	Standard error	95% CI low	95% CI high	VIF
Intercept	39.79	1	2.73	33.93	45.66	
A-Conc	− 8.11	1	1.76	− 11.89	− 4.32	1.0000
B-Time	6.41	1	1.76	2.63	10.20	1.0000
C-pH	14.21	1	1.76	10.42	17.99	1.0000
D-Dose	− 8.66	1	1.76	− 12.45	− 4.88	1.0000
AB	− 2.16	1	3.06	− 8.71	4.39	1.0000
AC	1.47	1	3.06	− 5.09	8.02	1.0000
AD	− 11.16	1	3.06	− 17.71	− 4.60	1.0000
BC	12.37	1	3.06	5.81	18.92	1.0000
BD	− 0.5050	1	3.06	− 7.06	6.05	1.0000
CD	− 2.57	1	3.06	− 9.13	3.98	1.0000
A^2^	10.83	1	2.40	5.68	15.98	1.08
B^2^	9.01	1	2.40	3.86	14.16	1.08
C^2^	10.58	1	2.40	5.43	15.72	1.08
D^2^	− 7.09	1	2.40	− 12.24	− 1.94	1.08

According to Table 4, the quadratic model for TTC adsorption based on the coded factors is presented in Eq. (13):

13 $$\begin{aligned} {\text{Y}} & = 39.79 - 8.11{\text{A}} + 6.41{\text{B}} + 14.21{\text{C}} - 8.66{\text{D}} - 2.16{\text{AB}} + 1.47{\text{AC}} \\ & \quad - 11.16{\text{AD}} + 12.37{\text{BC}} - 0.5050{\text{BD}} - 2.57{\text{CD}} \\ & \quad + 10.83{\text{A}}^{2} + 9.01{\text{B}}^{2} + 10.58{\text{C}}^{2} {-}7.09{\text{D}}^{2} \\ \end{aligned}$$

In this formula, each model has fixed and variable components. According to this, the predicted removal efficiency was 39.79%. The coded factors of A, B, C, and D had coefficients of − 8.11, + 6.41, + 14.21, and − 8.66, respectively. Also, the variable C, with a maximum coefficient of + 14.21 influenced the percentage of TTC adsorption. BC had the highest interaction with a coefficient of + 12.37, and A^2^ had the largest square effect among the factors with a coefficient of + 10.83.

Table 5 presents the analysis of variance (ANOVA) for the quadratic model of TTC removal using PVC. The values of R^2^, adjusted R^2^, predicted R^2^, and adequacy precision were obtained as 0.93, 0.87, 0.69, and 17.46, respectively. Generally, the findings in Table 5 were significant (*p* value < 0.05).

**Table 5:** ANOVA for the quadratic model of TTC removal by PVC.

	Sum of squares	*df*	Mean square	F-value	*p* Value
Model	8127.80	14	580.56	15.54	< 0.0001
A-Conc	788.62	1	788.62	21.11	< 0.0004
B-Time	493.31	1	493.31	13.20	< 0.0027
C-pH	2421.67	1	2421.67	64.82	< 0.0001
D-Dose	900.47	1	900.47	24.10	< 0.0002
AB	18.66	1	18.66	0.4995	0.4913
AC	8.61	1	8.61	0.2306	0.6385
AD	497.96	1	497.96	13.33	0.0026
BC	611.57	1	611.57	16.37	0.0012
BD	1.02	1	1.02	0.0273	0.8711
CD	26.52	1	26.52	0.7099	0.4136
A^2^	760.75	1	760.75	20.36	< 0.0005
B^2^	526.83	1	526.83	14.10	0.0021
C^2^	725.51	1	725.51	19.42	0.0006
D^2^	325.98	1	325.98	8.73	0.0105
Residual	523.05	14	37.36		
Lack of fit	436.47	10	43.65	2.02	0.2605
Pure error	86.58	4	21.64		
Cor total	8650.85	28			
R^2^	0.93		Predicted R^2^	0.69	
Adjusted R^2^	0.87		Adeq Precision	17.46	

**Figure 5:**
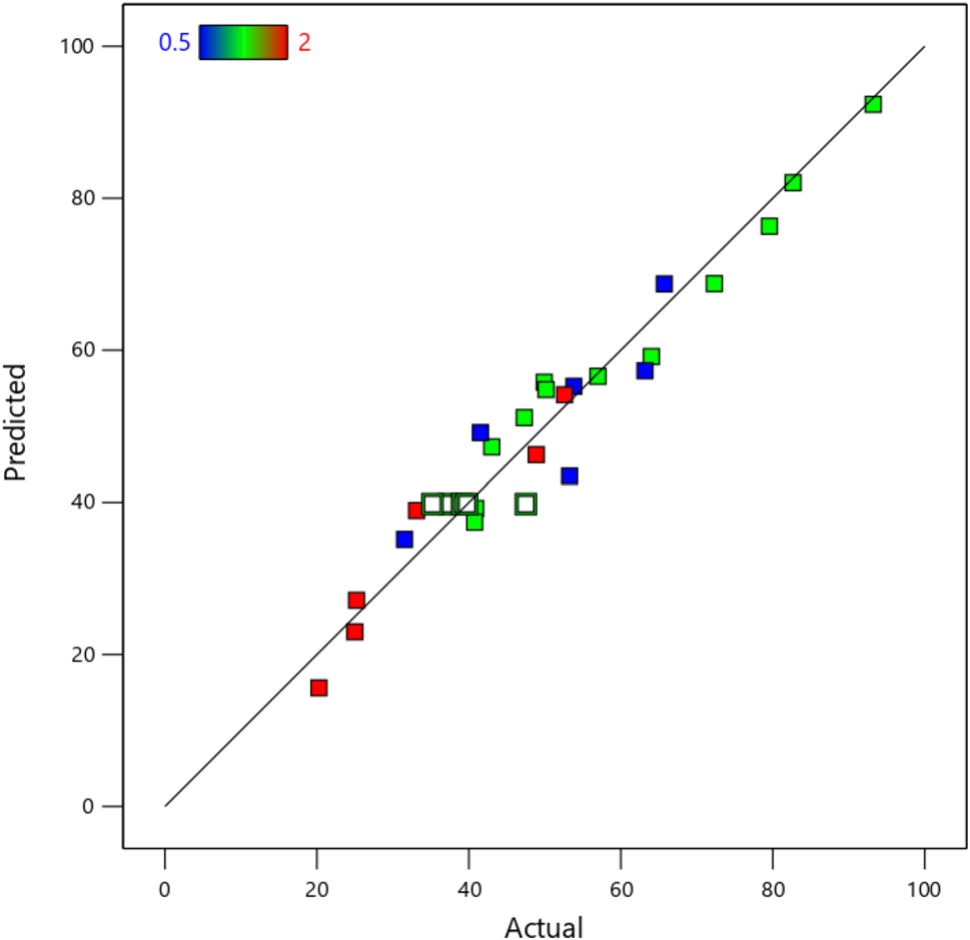
Distribution of experimental vs. predicted adsorption rates of TTC.

In Table 5, the *p* value for each main variable is below 0.05, indicating a statistically significant effect on the amount of TTC removal. Additionally, the difference between R^2^ and the predicted R^2^ is less than 0.2, which meets the recommended criteria for this model. Figure 5 illustrates the comparison between the actual removal of TTC and the predicted removal of TTC. Based on Fig. 5, it is clear that the model performs well in accurately predicting the removal of TTC.

### Impact of key factors on adsorption rate

**Figure 6:**
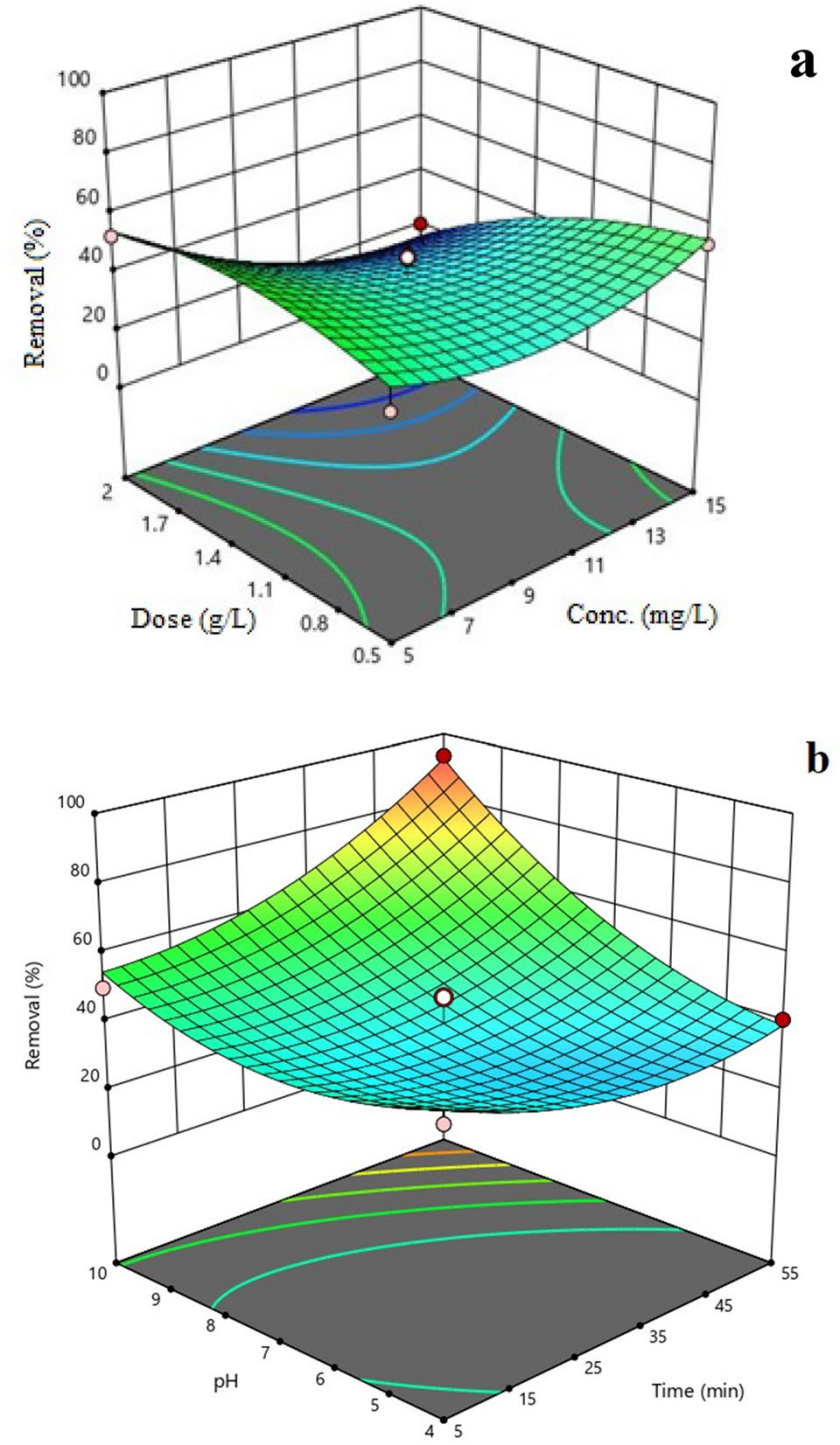
Response surface plots depicting the effects of dose versus concentration (**a**) and pH versus time (**b**).

Figure 6a,b illustrates the correlation between adsorption efficiency and various factors, including initial TTC concentration, contact time, pH, and PVC dose.

### Effect of initial TTC concentration

Pollutant concentration in the adsorption process is considered a determining factor in the transfer of pollutants between solid materials and the water environment. According to Fig. 6a, when the concentration was increased from 5 to 15 mg/L, the adsorption of TTC towards PVC decreased by 18.97%. This decreasing trend at higher concentrations can be attributed to the saturation of active adsorption sites on PVC. Basically, when the concentration of antibiotics increases, there is intense competition among the antibiotic molecules to bind to the adsorbent surface. As a result, not all molecules can adhere effectively to the adsorbent surface, leading to a decrease in removal efficiency. In addition, the presence of antibiotic molecules on the adsorbent creates a repulsive force that prevents the adsorption of other antibiotic molecules onto PVC^42,43^.

### Effect of adsorbent dose

Examining the adsorbent dose is important because it has numerous hollow active sites for adsorbing pollutants. According to Fig. 6a, when the adsorbent dose was increased from 0.5 to 2g/L, the adsorption rate decreased by 41.36% (*p* value < 0.05). In fact, the optimal dose for achieving the highest adsorption rate of TTC is 0.5 g/L of adsorbent. The low adsorption rate at high adsorbent dosage may be attributed to the fact that, in some cases, a high dose of adsorbent can result in limitations in pollutant transport. In fact, at high doses, the adsorbent particles can form aggregates or clumps, which can hinder the movement of the antibiotic to the adsorption sites. This reduces the contact between the adsorbent and the antibiotic, thereby decreasing the rate of adsorption. At low adsorbent doses, there is a lower possibility of adsorbent accumulation and a higher adsorption of TTC molecules towards the adsorbent surface^44,45^.

### Effect of contact time

According to Fig. 6b, contact time had a direct effect on TTC adsorption (*p* value < 0.05). According to the results obtained from this study, the optimal duration of conversation was found to be 55 min. Accordingly, by increasing the contact time from 5 to 55 min, the adsorption efficiency increased by 12.82%. Over time, there is an increasing opportunity for contact between antibiotic molecules and MPs. As a result, PVC adsorbs more TTC molecules. The results of the study conducted by Mirslimani et al. (2018) on MOF-5 is consistent with the findings of our study^17^.

### Effect of pH

The pH of the solution is one of the factors that determine the mechanism of TTC adsorption onto PVC. By influencing the surface charge of PVC, this parameter impacts the electrostatic interaction between the surface and the antibiotic, thereby affecting the overall performance of the adsorption process. The results of Fig. 6 show that the rate of TTC adsorption increases with increasing pH (*p* value < 0.05). As the pH increases, the surface of PVC becomes more negatively charged. Consequently, the antibiotic is adsorbed to PVC through electrostatic interaction. The isoelectric point is an important parameter because it affects the behavior of the adsorbent and the electrostatic interaction^46^. Based on the results, the pH of the isoelectric point was found 6.65 that confirms the mentioned contents. Therefore, it can be concluded that the surface charge of PVC is positive below the isoelectric point and negative above it^47^.

### Kinetic and isotherm models

Adsorption isotherms can evaluate the adsorption performance of pollutants by assessing the distribution of saturated molecules between the liquid and solid phases until equilibrium is reached^45^. In this research, the adsorption performance of TTC was investigated using the Temkin, Langmuir, and Freundlich isotherm models. Table 6 shows the kinetic and isotherm parameters for TTC adsorption by PVC. The results of Table 6 showed that the adsorption of antibiotics by PVC follows the Langmuir model. Tang et al.^48^, who investigated the adsorption of antibiotics by MPs obtained similar results to our study. Kinetics provides important information about the relationship between time and mass transfer of the adsorbent in the adsorption process. In addition, it shows adsorption mechanisms, including chemical reaction and diffusion. In fact, collecting this information is useful for understanding the adsorption mechanism^49^. Pseudo-first-order, pseudo-second-order kinetic models, and interparticle penetration kinetics are commonly considered models of adsorption kinetics. From Table 6, the (R^2^) values for pseudo-first-order kinetics, pseudo-second-order kinetics, and intraparticle diffusion kinetics were 0.87, 0.98, and 0.42, respectively. Therefore, the kinetics of TTC uptake by PVC follows a pseudo-second-order model. Debnath B (2020) obtained similar results on the adsorption of TTC by zirconia^42^.

**Table 6:** Kinetic and isotherm parameters fitted for TTC removal using PVC.

Kinetic model	Linear form	Parameter	Value			
			2 mg.L^−1^	4 mg.L^−1^	8 mg.L^−1^	16 mg.L^−1^
Pseudo-first order	$\log \left( {q_{e} - q_{t} } \right) = logq_{e} - \frac{{k_{1} }}{2.303} \cdot t$	q_e,cal_ [mg/g]	4.01	5.98	2.85	2.97
		K_1_ [min^−1^]	0.19	0.02	0.14	0.13
		R^2^	0.10	0.50	0.87	0.33
Pseudo- second order	$\frac{{\text{t}}}{{{\text{q}}_{{\text{t}}} }} = \frac{1}{{k_{2} q^{2} }} + \frac{1}{{q}} \cdot {\text{t}}$	q_e,cal_ [mg/g]	17.5	97.61	41.83	28.34
		K_2_ [min^−1^]	27.33	15.18	65.07	44.08
		R^2^	0.91	0.91	0.91	0.98
Intra-particle diffusion	$q{\text{t}} = k_{p} \cdot t^{0.5} + c$	K_p_ [mg/g min^−0.5^]	0.014	0.02	0.06	0.08
		R^2^	0.42	0.42	0.42	0.42

Isotherm model	Linear form	Parameter	Value			
Langmuir	$\frac{{{\text{Ce}}}}{{{\text{qe}}}} = \frac{1}{{{\text{qm}}}}{\text{Ce}} + \frac{1}{{{\text{qmb}}}}$	q_max_ (mg/g)	21			
		K_L_ (L/mg)	0.04			
		R^2^	0.98			
Freundlich	Log $q_{e}$ = log $K_{F}$ + $\frac{1}{n}$ log $C_{e}$	K_F_ mg/g(L/mg)^1/n^	1.23			
		n	4.03			
		R^2^	0.81			
Temkin	$q_{e} = B_{1} ln \cdot k_{t} + B_{1} lnC_{e}$	k_t_ (L/mg)	31088.91			
		B_1_	0.0081			
		R^2^	0.83			

### Effect of thermodynamic

In order to accurately investigate the effect of temperature on the amount of adsorption and to determine whether the TTC adsorption process is exothermic or endothermic, thermodynamic studies were conducted under optimal laboratory conditions. Thermodynamic data for the adsorption of TTC to PVC are presented in Table 7. As can be seen from Table 7, ΔG° in the present study is negative, which indicates that the adsorption process is favorable and spontaneous. In addition, the positive value of ΔS° is due to the increase in disorder during the adsorption process at the liquid–solid interface. The obtained ΔH° value is between 1 and 93 kJ/mol, indicating that the adsorption mechanism follows physical adsorption^50,51^. The negative values of ΔH° indicate the exothermic nature of the adsorption process, which leads to a decrease in adsorption efficiency as temperature increases. Findings indicated that the adsorption of TTC at 273 °K is higher than at other temperatures. In general, it can be concluded that the removal efficiency decreases as the temperature increases. The decrease in efficiency at high temperatures may be attributed to changes in the structure of the adsorbent. These changes can increase the movement of antibiotic molecules and their tendency to separate from the absorbent surface. Additionally, the hydrogen bond between TTC and PVC may be broken^52^. The results of this study are consistent with the findings of Bao et al.^53^, who investigated the adsorption of phenanthrene and its monohydroxy derivatives on PVC MPs.

**Table 7:** Thermodynamics parameters for TTC adsorption onto PVC.

T (K)	Adsorption rate (%)	ΔG° (kJ/mol)	ΔH° (kJ/mol)	ΔS° (J/(mol K)
298	94.68	− 4759.10	− 8.3	25.77
308	52.63	− 3562.48		
313	34.5	− 1926.39		
318	32.16	− 1744.29		

### Effect of ions

**Figure 7:**
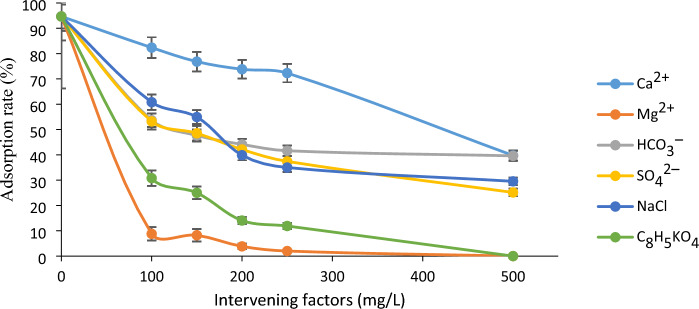
Effect of different ions on TTC adsorption.

Interfering compounds were also considered as part of this study. The presence of these compounds in water can either decrease or increase the adsorption rate of TTC. In this study, we investigated the effect of compounds such as CaCO_3_, MgSO_4_, NaHCO_3_, Na_2_SO_4_, C_8_H_5_KO_4_, and NaCl on the adsorption rate of TTC. Figure 7 investigates the adsorption rate in the presence of various ions at concentrations ranging from 100 to 500 mg/L. Figure 7 shows that the adsorption rate of TTC decreases as the concentration of the interveners increase. Accordingly, the presence of magnesium ions and the compound C_8_H_5_KO_4_ at a concentration of 500 mg significantly reduces the adsorption rate of TTC compared to other interfering factors, ultimately resulting in a complete adsorption rate of zero. Ca^2+^ and Mg^2+^ ions can compete with larger pollutants (such as TTC molecules) to be adsorbed onto the PVC surface. As a result, these ions are adsorbed faster than TTC, leading to a decrease in the efficiency of the TTC adsorption process. Indeed, Ca_2_^+^ and Mg_2_^+^ ions have a suppressive effect on TTC uptake in MP^52^. On the other hand, anions such as HCO_3_^−^ and SO_4_^2−^ may lead to a decrease in adsorption capacity by forming a hydrophobic complex with TTC^39,54^. NaCl is the most common compound in the water environment and has a significant effect on the adsorption of antibiotics by MPs. With increasing salinity, Na^+^ cations easily bind to negatively charged MPs through electrostatic interaction, preventing the adsorption of TTC by MPs^3^. Furthermore, by adding potassium hydrogen phthalate (C_8_H_5_KO_4_) to the solution, the concentration of H^+^ ions in the solution increases, resulting in a decrease in adsorption efficiency. This may be due to changes in the pH of the solution. Li et al.^9^ demonstrated that the adsorption of antibiotics on MPs in a seawater system decreased in comparison to a freshwater system, primarily due to the elevated salinity. Also, Guo et al.^55^ reported that the adsorption capacity of sulfamethazine on PE, PVC, and PP decreased with increasing salinity due to electrostatic interactions.

### Field studies

**Figure 8:**
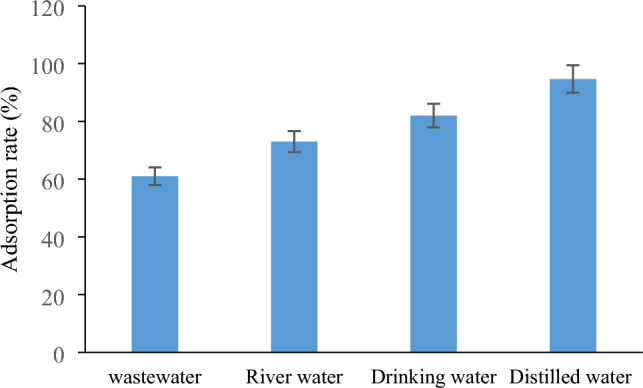
Effect of different water resource on TTC adsorption.

To conduct this test, TTC was initially added to the samples under study at a concentration of 5 mg/L. Then, the adsorption rate of TTC onto PVC was measured in various water environments. Figure 8 shows the effect of different water environments on TTC adsorption. For this purpose, samples of distilled water, drinking water, river water, and urban sewage were used. The electrical conductivities (ECs) of these samples were 0.001, 0.5, 1.49, and 3.49 mS/cm, respectively. As depicted in Fig. 8, the TTC had the highest adsorption rate in distilled water (94.68%), whereas the lowest removal efficiency was observed in municipal wastewater (61%). Considering that distilled water has the lowest EC and urban wastewater has the highest EC, it can be concluded that the ions present in these water environments acted as an intervention and reduced the adsorption rate of TTC^56^.

## Conclusion

In this study, the optimization of TTC adsorption by PVC was done using the Box-Benken model. The maximum adsorption rate (93.23%) was obtained at a pH of 10, a contact time of 55 min, an adsorbent dose of 1.25 g/L, and a TTC concentration of 10 mg/L. The findings followed the pseudo-second-order model and the Langmuir isotherm model. TTC was adsorbed onto PVC through polar-polar interactions and hydrogen bonding. According to thermodynamic findings, the process mechanism was spontaneous, exothermic, and physical. By examining the effect of ions, it was found that the adsorption process decreases with an increasing concentration of ions. The results showed that the adsorption of TTC was minimal in urban wastewater and maximal in distilled water. This study provides insight into the mechanisms and main factors that influence the environmental behavior of antibiotics and MPs. This information is crucial for controlling and assessing the risks associated with them.

## Data Availability

The datasets generated and analyzed during the current study were available from the corresponding author on reasonable request.
